# Vivisection

**Published:** 1892-02-27

**Authors:** Lester Reed

**Affiliations:** Public Analyst for the Borough of Croydon. 19, South Park Hiil Road, Croydon


### EDITOR'S LETTER-BOX.

[Our correspondents are reminded that prolixity is a great bar to publication, and that brevity of style and conciseness of statement greatly facilitate early insertion.]

### VIVISECTION.

Sir,—As I notice that in your issue of December 26th the vivisection question is alluded to as one at which it is desirable to look "in the light of every variety of intelligence," perhaps you will not be altogether unwilling to allow some little space in your paper for the consideration of it. In defence of the practice one may point first to the scientific facts (and every fact is a scientific fact) which appear to have been either disclosed or disseminated by its means; secondly, it is defended by some on the ground that they believe it to be possible to restrain the practice within that undefinable limit, the existence of which is admitted by every thoughtful and merciful man, and, I believe, often more readily by those who have some practical acquaintance with vivisection than by those who are altogether outsiders; beyond which undefinable boundary, not only does the practice become ethically unsound, but on account of this ethical unsoundness becomes also an indirect, but not, therefore, a less real or to be dreaded, danger to society; thirdly, vivisection is often defended from the analogies of Nature, some of which, taken by themselves, would seem to point to the almost absolute right of might. To the medical mind, or, indeed to the mind of any man acquainted with the details of practical experimental physiology, either from personal experience or otherwise, the popular distinction between "unnecessary cruelty" and such experiments as are "medically necessary" can convey no absolute meaning whatever. Such a distinction may exist, and may appear perfectly clear in the minds of those who only talk about vivisection; but to the workers in a physiological laboratory, its visionary and vague nature must be apparent. Between the decapitation of a frog with momentary pain, for purposes of the highest scientific imtance on the one hand, and, on the other, the works of such a vivisector as the poet Browning's monster "who would have no end of brutes cut up alive to save his toe from shoots," there must be vivisections of every degree of uselessness and usefulness on the one hand, and of painfulness and painlessness on the other, unless indeed, assuming vivisection to be unsound in principle morally, we are to expect, as some do, what would really amount to "a sign from Heaven," in the shape of the immediate failure of the practice for this reason alone. So that it becomes evident that no attempt to classify vivisections accurately into "unnecessary" or "useless" and "necessary" or "useful" can be satisfactory, while, on the other hand, in a question relating directly to oppression, we are, I think, shirking a very serious responsibility, if we regard the matter as merely a question for the individual conscience. If then there be two sides to this question, it must be argued between general principles rather than with respect to details, and we have to decide whether the principles upon which vivisection is held to be justifiable as a system of research, tend more, or tend less, to the ultimate stability of manners and of society than do those upon which it is held to be unjustifiable. Every law of conduct which is to have any permanent force at all can only retain this force so long as it is believed to be the expression of an eternal principle, or of what we may term a truth in the imperative mood. Directly those rules, by the observance of which society subsists, cease to be believed to be this, their force begins to degenerate until it becomes merely that of the policeman's cudgel and the hangman's rope. Can society afford to drop, or even to weaken in any degree, its belief in the inviolable rights of individuals? If not, then surely it is in the highest degree inexpedient to support a practice the justification of which is based upon the denial of the principle of inviolable rights. I well know that the definition of these rights, especially in the case of the lower animals, is encompassed with difficulties; but while the testimonies alike of science and of religion unitedly point to a great difference in importance or value between the lives of animals and those of men, we have no ground whatever for extending this difference of value to pain, and therefore we cannot assert the right of the human individual to personal and abstract justice in respect of pain unless we also assert the same in the case of those animals whose capacity for suffering is probably not far inferior to our own, while it will be borne in mind that the vivisection question is not merely one of relative quantities of pain, but is rather a question of justice in the infliction of pain by moral agents. Nobody doubts that the human race may be benefited in many other ways than by the prolonged torture of innocent victims, and that, moreover, without the danger of inculcating or sanctioning principles subversive in their ultimate working of personal confidence in our fellow men; and so long as this remains the case it must at least be a question worthy of our very serious consideration, whether it would not be in every way better and safer to direct our efforts for the good of humanity exclusively into those other equally workable and probably inexhaustible channels from which this danger, at any rate, is manifestly absent.—Yours, &c.

Lester Reed, F.C.S., F.I.C.

Public Analyst for the Borough of Croydon.

19, South Park Hill Road, Croydon.

